# Heavy Metal Uptake by Herbs. IV. Influence of Soil pH on the Content of Heavy Metals in *Valeriana officinalis* L.

**DOI:** 10.1007/s11270-015-2360-3

**Published:** 2015-03-21

**Authors:** Dorota Adamczyk-Szabela, Justyna Markiewicz, Wojciech M. Wolf

**Affiliations:** 1 Institute of General and Ecological Chemistry, Łódź University of Technology, Żeromskiego 116, 90-924 Łódź, Poland; 2 Present Address: Department of Molecular Engineering, Faculty of Process Engineering and Environmental Protection, Łódź University of Technology, Wólczańska 213, 90-924 Łódź, Poland

**Keywords:** *Valeriana officinalis*, Soil, Heavy metals, Herbs, FAAS

## Abstract

The aim of the study was to estimate the influence of soil pH on the uptake of copper, zinc, and manganese by *Valeriana officinalis*. Preliminary studies involved soil analyses to determine acidity, organic matter content, and copper, zinc, and manganese total and bioavailable forms. The study involved atomic absorption spectrometry to determine the concentration of the elements, and mineral soil of pH = 5.1 was used in the study, as being typical for central Poland. The copper, zinc, and manganese contents were determined in plants grown in soils which had been modified to cover a wide range of pH values 3÷13. The intensity of germination was strongly pH dependent with the highest yield obtained in original, unmodified soil. Surprisingly, high soil alkalinity stimulated copper and manganese uptake while at the same time resulting in a decrease in zinc content.

## Introduction

Herbs, which have always been the principal form of medicine in developing countries, are once again becoming popular throughout the world, in developing and developed countries alike, with statistics showing that a growing number of people in Europe, North America, and Australia are consulting professional herbalists (Cordell and Colvard 2012; Kumar 2006).

In recent years, much research has been aimed at drugs of plant origin. The World Health Organization estimates that 80 % of the world’s population relies on herbal medicine (Cass 2004). In particular, herbal therapies are commonly used for treatment of chronic symptoms of sleep disturbances (Taibi et al. 2007), as this common syndrome affects an ever increasing proportion of the human population. For example, almost one third of all adults in the USA report various symptoms of insomnia (Aikens and Rouse 2005; Barnes et al. 2008; Grewal and Doghramji 2010).

*Valeriana officinalis* L. (valerian) is a perennial herbaceous plant (Valerianaceae family) that has been widely used in therapy since ancient times and is nowadays widely distributed and cultivated across Europe and Asia, as native there. In Poland, it is one of the major herbs processed by the pharmaceutical industry, with a total annual harvest exceeding 1000 t (Baj 2010).

Valerian root extract demonstrates strong sedative properties, and in the USA and many European countries including Poland, it is used to alleviate states of excessive nervous agitation, anxiety, difficulty with concentration, or insomnia (Bent et al. 2006; Sudati et al. 2009; Weeks 2009). In particular, the valeric acid present in *V. officinalis* exhibits spasmolytic action and reduces tension in gastrointestinal smooth muscles (Wielgosz et al. 2012). The health properties of valerian are fairly well documented (Ghedira et al. 2008; Patocka and Jakl 2010). The major active compounds are essential oil, valepotriates, and valeric acids with derivatives, although the detailed mechanism of their action is not yet fully understood (Baranauskiene 2007). This situation has prompted studies in biology and medicine of the valerian plant accompanied by identification of its active chemical substances (Erns 2006; Ozkol et al. 2011; Stef et al. 2010; Sudati et al. 2009; Wang et al. 2011). Experimental cultivation conditions as described in the present paper were based on a general knowledge of the *V. officinalis* biology. It is well recognized that this herb prefers moist woodlands and is often planted on a low lying damp sandy humus with possible addition of lime fertilizer (Patocka and Jakl 2010; Bernath 1997).

It is well documented that different conditions of valerian cultivation may cause substantial changes in the chemical constitution of the plant as well as in its medical effects (Tabatabaei 2008). The latter may be also affected by the processing method and storage conditions of the herbs harvested (Kemper 1999; Peirce 1999). In the case of medical products, it is particularly important to control the content of heavy metals accumulated during the plant’s growth (WHO 2007; Leśniewicz et al. 2006; Bu et al. 2013). Herbs cultivated on soils with high content of heavy metals often show plant stress symptoms (Ovečka and Takáč 2014; Viehweger 2014) and subsequent variations in plant constituents. This may obviously influence the medical value of particular species. European and Polish domestic regulations are restricted to cadmium, lead, and chromium (WHO 2007; Pharmeuropa 2008; Regulation of the Minister of Health 2003) only. However, many other heavy metals also introduce plant stress, among which zinc, manganese, and copper are the most important (Cheng 2003; Farrag et al. 2012).

Due to the varied bioavailability of heavy metals in soil, their concentration in herbs available on the market may vary significantly (Kalny et al. 2007; Kandziora-Ciupa et al. 2013). Heavy metal uptake by plants is a complex soil-plant process, influenced by many factors such as plant species, genotype, availability, and mobility of metals in soil, soil properties, and all the biogeochemical processes and the microbial activity, at the rhizosphere level, which influence metal mobility and availability to plants (Gębski 1998; Kabata-Pendias and Pendias 1999; Radanovic et al. 2002; Sady and Smoleń 2004; Farrag et al. 2012; Nadgórska-Socha et al. 2013). The latter were studied in detail, and there is a common understanding reported in the literature that soil pH is the major factor influencing the mobility and bioavailability of heavy metals to plants (Cheng 2003; Domańska and Filipek 2011; Ginocchio et al. 2002; Kukier et al. 2004; Pikuła and Stępień 2007; Wang et al. 2006). Generally, the mobility of metals in alkaline soils decreases in the order Cd > Ni > Zn > Mn > Cu > Pb (Fijałkowski et al. 2012) and is highly variable and strongly dependent on the content and type of organic ingredients as present in the soil (Badaway et al. 2002; Braillier et al. 1996; du Laing et al. 2008; Wang et al. 2006). In particular, soils with pH values below 7 are very prone to heavy metal migration from soil solid components into the soil solution, as has been observed for copper (Wang et al. 2006; Zeng et al. 2011), manganese (Alam et al. 1999), and zinc (Bang and Hesterbery 2004; Marschner 1993).

Herbal therapies are usually long term. Therefore, even small heavy metal doses as present in particular plant may accumulate in patient body over a long period of time (Aikens and Rouse 2005; Kraft and Hobbs 2004).

In this paper, the influence of soil pH on the uptake of selected heavy metals by valerian plants cultivated in the model pot experiments was investigated. This work follows our ongoing investigations on the influence of cultivation conditions and methods on the heavy metal uptake by herbs (Adamczyk 2006; Adamczyk 2007; Adamczyk and Jankiewicz 2008).

## Materials and Methods

Soil samples were collected in October 2011, in Krokocice village (Szadek municipality, Łódź province, about 30 km from Łódź, Poland), on agricultural land located away from excessive traffic according to the procedure in PN-ISO 10381-4:^2007^. The samples were subsequently dried in a well-ventilated place, sifted through a 2-mm stainless steel sieve, and finally stored in plastic bags. Soil pH was measured by the potentiometric method in 1 mol/dm^3^ potassium chloride solution (PN-ISO 10390:^1997^). The well-established gravimetric method for the determination of soil organic matter by the mass loss at 550 °C was applied (Nelson and Sommers 1996; ASTM 2000; Schumacher 2002). The bioavailable forms of metals were determined in 1 mol/dm^3^ hydrochloric acid extracts (PN-ISO 11259:^2001^). The total metal content was measured in samples mineralized using the Anton Paar Multiwave 3000 closed system instrument. The mixture of concentrated HNO_3_ (6 cm^3^) and HCl (2 cm^3^) was applied (0.5 g of soil). Metal concentrations were measured by FAAS with the GBC 932 plus spectrometer. The results are summarized in Table 1.

**Table 1:** Results of soil analysis

Analysis	Results	
Soil pH	5.1	
Organic matter	3.33 %	
Metal content	Total (mg/kg)	Bioavailable (mg/kg)
	Copper	
	4.40 ± 0.44	1.23 ± 0.16
	Zinc	
	23.3 ± 1.9	3.63 ± 0.41
	Manganese	
	253 ± 15	83.6 ± 1.5

*n* = 4; *p* = 95 % (*n*, number of sample; *p*, confidence level)

### Soil pH Adjustment and Preparation of Plant Samples

Soil pH was originally adjusted to four values: 3.5, 4.1, 10, and 13. Samples of agricultural soil (500 g) were placed in 16 plastic pots (4 for each pH value), and then appropriate amounts of 0.5 mol/l H_2_SO_4_ (30 or 125 ml) or CaO (8 or 16 g) were added to each sample and carefully mixed manually. The soil with additives prepared in this way was subsequently air-dried and subjected to pH measurements. The whole procedure was consequently repeated until the final pH value was established. For the plant growth, 40 plastic pots were used (4 for each pH value and 4 extra representing additive-free soil).

The weight of soil sample used per pot was the same as in the pH adjustment (500 g). Seeds of *V. officinalis* (from P.H. Legutko company, Poland) were sown in an amount of 0.2 g (approximately 200 seeds) per pot. All pots were kept in a greenhouse at a controlled temperature of 23 ± 2 °C. All plants were regularly watered by deionized water and soil moisture was kept at 60 % of its maximal water holding capacity. The *V. officinalis* was harvested 3 months after they were sown. After harvesting, the plants were washed several times in deionized water and oven-dried at 45 °C to a constant weight. The dried valerian plant roots and seeds were subjected to a microwave mineralization process under concentrated HNO_3_ (6 cm^3^) and HCl (1 cm^3^) (0.5-g sample). The zinc, manganese, and copper concentrations in the resulting solutions were determined by FAAS with the GBC 932 plus spectrometer.

The accuracy of the method applied was controlled by parallel analysis of the certified reference material INCT-MPH-2, containing a mixture of selected Polish herbs. Results are collected in Table 2. Data were statistically evaluated using a one-way ANOVA as implemented in the Microsoft Excel 2010.

**Table 2:** The content of Cu, Zn, and Mn in the certified reference material

Metal	Certified value (mg/kg)	Found (mg/kg)	Recovery (%)
Copper	7.77 ± 0.53	6.93 ± 0.38	89
Zinc	33.5 ± 2.1	34.5 ± 0.9	103
Manganese	191 ± 12	179 ± 9	94

*n* = 4; *p* = 95 % (*n*, number of samples; *p*, level of confidence)

## Results

The results of the soil analysis and metal bioavailability are summarized in Table 1. The organic matter content was determined as 3.33 %, which indicates the mineral character of the soil (Fotyma and Mercik 2003; Dobrzański and Zawadzki 1995), while the low copper, zinc, and manganese content clearly indicates that, according to the generally accepted international standards (Council Directive 86/278/EEC 1986; IUSS Working Group WRB 2006), the soil is not contaminated by these metals. The results for the metal content in the valerian roots and seeds are summarized in Table 3. Results of ANOVA clearly showed that variations of metal contents as calculated for herbs grown in soils of pH values 5.1 and 10.0 are significantly different (Table 4).

**Table 3:** Selected metal content accompanied by average dry plant weights and germination rates as determined for *Valeriana Officinalis*

Cultivation conditions	Germination rate (%)	Average dry plant weight^a^ (g)	Average dry roots weight^b^ (g)	Metals content in roots (mg/kg)		
				Copper	Zinc	Manganese
pH = 5.1	34	1.68 ± 0.42	0.62 ± 0.32	8.50 ± 1.20	49.5 ± 4.1	81.0 ± 2.1
pH = 10	11	0.40 ± 0.17	0.26 ± 0.11	15.0 ± 1.5	26.2 ± 1.7	106 ± 2

^a^Average dry plant weight as calculated for a single plant (g)

^b^Average dry root weight as calculated for a single plant (g)

**Table 4:** ANOVA parameters for metal content in *Valeriana officinalis* across two soil pH values

Source of variation	SS	MS	F	*P* value	Test F
Zinc	1092.781	1092.781	109.0283	4.52 · 10^−5^	13.745
Copper	84.43501	84.43501	52.62893	0.000349	13.745
Manganese	1225.125	1225.125	230.7928	5.13 · 10^−6^	13.745

**Fig. 1:**
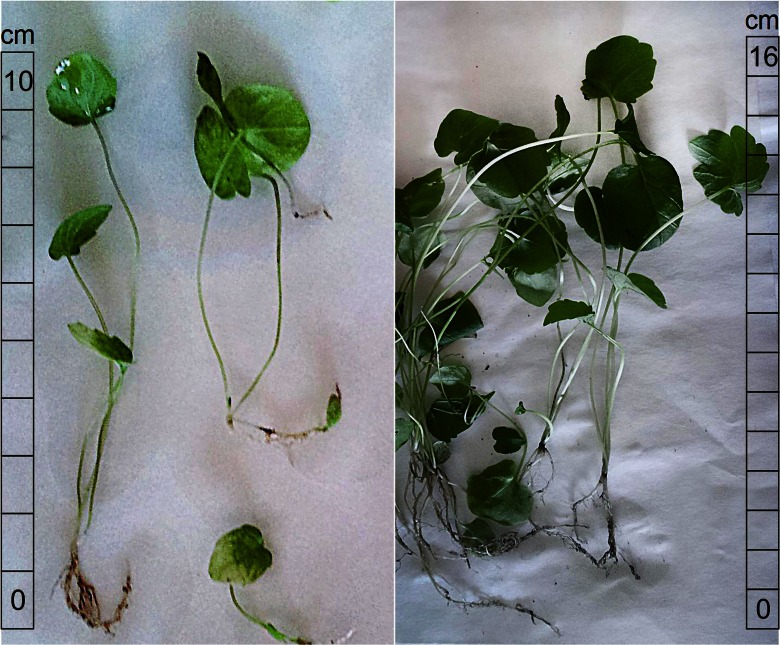
Plants cultivated on soil with pH = 5.1 and pH = 10 after harvest

The most intensive germination and plant growth was observed at pH = 5.1. Increased alkalinity to pH = 10 significantly inhibited both the seed germination and the plant growth (Fig. 1). Despite several attempts, valerian seeds inoculated in soil characterized by a low or a very high pH values (3.5, 4.1, 13.0) failed to germinate. Metal content in seeds was 11. 5 ± 1.5, 37.2 ± 0.6, and 63.6 ± 2.2 mg/kg for copper, zinc, and manganese, respectively.

## Discussion

It has been widely documented that heavy metal uptake by plants from soil is strongly pH dependent (Bolan et al. 2003; Chairidchai and Ritchie 1993; Hollier and Reid 2005; Wang et al. 2006), and this conclusion was further confirmed by the present paper. Increasing the soil pH to 10 resulted in a significant decrease in the zinc content of all plants investigated (Table 3). This is quite consistent with numerous literature data, which indicate that at high soil alkalinity, plants are not prone to zinc uptake (Bang and Hesterberg 2004; Kukier et al. 2004; Radanovic et al. 2002; Sukreeyapongse et al. 2002; Tyler and Olsson 2001; Wang et al. 2006; Zeng et al. 2011). Valerian seeds did not germinate in the soil of pH value below 4.5; therefore, it was not possible to determine the metal uptake in these extremely acidic conditions. However, there is a general understanding that low pH prompts high metal mobility (Reddy et al. 1995; Landner and Reuther 2005; Violante et al. 2010). In particular, Pikuła and Stępień (2007) pointed out that the significant zinc bioavailability in acidic conditions may be related to the formation of soluble mineral entities further releasing metal ions into the soil environment. Surprisingly, copper and manganese contents in herbs grown in highly alkaline soil (pH = 10) are significantly higher as compared to plants cultivated in medium acidic conditions (pH = 5.1). Increasing the soil pH does not necessarily reduce the amount of bioavailable heavy metals (Wu et al. 2011). According to Kabata-Pendias and Pendias (1999), mobility of these metals in alkaline soils is often increased due to the formation of complexes involving the soil organic entities which are available for plants (Gębski 1998; Majewska and Kurek 2002). It is quite well documented that pH and the redox conditions influence manganese bioavailability in soils (Marschner 1995; Porter et al. 2004). In acidic soils (pH < 5.5) additionally characterized by high redox potential (Kogelmann and Sharpe 2006), manganese oxides are easily reduced to Mn^2+^ ions which are available for plants (Adriano 2001; Watmough et al. 2007). At high soil pH (pH > 8.0), chemical Mn^2+^ auto-oxidation may lead to the formation of MnO_2_, Mn_2_O_3_, Mn_3_O_4_, or even Mn_2_O_7_, which are not easily accessible to plants (Ducic and Polle 2005; Gherardi and Rengel 2004; Humpries et al 2007). Furthermore, these oxides can readily absorb on soil particles, further decreasing the manganese bioavailability (Fageria et al. 2002).

On the contrary, several reports have suggested that available forms of manganese may be produced under reduced conditions, even at high soil pH values (Hue 1988). In a similar way to the experimental setup as presented in this paper, a reducing environment existed when an excess of water in soil was combined with good availability of organic matter (El-Jaoual and Cox 1998). The latter interacted with solid manganese oxides transforming them into soluble complexes easily available for plants (Millaleo et al. 2010). It is also quite well documented that increasing pH, induced by lime, intensifies microbiological processes in soil (Fuentes et al. 2006; Weyman-Kaczmarkowa and Pedziwilk 1999c). Weyman-Kaczmarkowa and Pedziwilk (2000) have reported that lime alkalization prompts bacterial growth, especially in loose sandy or sandy loam soils. The resulting increase in microbial biomass concentration accumulated considerable amounts of heavy metals (Bergman et al. 1999; Bewley and Stotzky 1983; Peick et al. 1999). The latter mechanism is presumably responsible for the high manganese and copper uptake by valerian plants as observed in the present study.

Our future research will concentrate on detailed mechanism of heavy metal uptake by *V. officinalis* cultivated in variable soil environments relative to the growing importance of herb production for the domestic and regional economics.
